# Genome‐wide identification of quantitative trait nucleotides for plant architecture‐related traits in peanut

**DOI:** 10.1002/tpg2.70119

**Published:** 2025-11-03

**Authors:** Juan Wang, Dachuan Shi, Cuiling Yuan, Yifei Mou, Haocui Miao, Yuan Li, Shihua Shan

**Affiliations:** ^1^ Shandong Peanut Research Institute Qingdao China; ^2^ Qingdao Academy of Agricultural Sciences Qingdao China; ^3^ Institute of Crop Germplasm Resource Xinjiang Academy of Agricultural Sciences Urumqi China; ^4^ Computational Biology and Biological Physics, Astronomy and Theoretical Physics Lund University Lund Sweden

## Abstract

Peanut (*Arachis hypogaea* L.) is globally recognized as an important oilseed crop. Traits related to plant architecture are closely associated with yield in peanut. In this study, we focused on four specific traits related to plant architecture—first branch length (FBL), main stem height (MSH), stem diameter (SD), and the number of nodes on the main stem (NSK)—across three locations. Using whole‐genome resequencing data from a genetically diverse collection of peanut landraces, we conducted a genome‐wide association study analysis to identify genetic variants associated with these traits. Notably, a novel genomic region on Arahy.03:39916768–42652757 was associated with SD for the first time. Homology analysis suggested that two annotated genes within this region may contribute to stem elongation and seed development. For MSH, NSK, and FBL, more than half of the significantly associated single‐nucleotide polymorphisms (SNPs) were localized on chromosome Arahy.05. Two SNPs at Arahy.09:112028951 and Arahy.09:112272948 were identified as the potential diagnostic markers for MSH and FBL: one homologous gene near these SNPs encoded an E3 ubiquitin–protein ligase, while the other encodes cinnamyl alcohol dehydrogenase. Additionally, one SNP at Arahy.05:53493734 was identified as a potential diagnostic marker for MSH, FBL, and NSK and validated using the penta‐primer amplification refractory mutation system and quantitative real‐time polymerase chain reaction. A gene near this SNP belongs to the protein kinase superfamily. Enzymes are known to regulate diverse cellular and biological processes, including plant development. These findings advance our understanding of the genetic basis of peanut architecture and provide valuable markers for future yield improvement efforts.

## Core Ideas

- We identified 411 significant single‐nucleotide polymorphisms (SNPs) associated with four traits related to peanut architecture through a genome‐wide association study.
- For the trait of stem diameter, we identified a novel genomic region on Arahy.03:39916768–42652757, associated with this trait for the first time.
- Two SNPs located at Arahy.09:112028951 and Arahy.09:112272948 were identified as the potential diagnostic markers for main stem height (MSH) and first branch length (FBL).
- An SNP located at Arahy.05:53493734 was associated with MSH, FBL, and the number of nodes on the main stem. The genomic region surrounding this SNP has been annotated as part of the protein kinase superfamily, which plays a crucial role in plant development.

## Plain Language Summary

The yield of peanut (*Arachis hypogaea* L.) is closely linked to its structural characteristics, which can significantly influence planting density and lodging resistance. Here, we examined the complete genomic sequence of the peanut to identify minor variations that may correlate with peanut architecture. We specifically analyzed the genome to uncover insights related to four specific traits: first branch length (FBL), main stem height (MSH), SD, and number of nodes on the main stem. Notably, some of these loci were associated with multiple traits, particularly MSH and FBL, corroborating findings from previous studies. One of our most exciting discoveries was three genomic loci that may serve as a reliable predictor for plant architecture. Furthermore, we identified a crucial segment and two candidate genes on Arahy.03 that appear to be linked to stem thickness. Gaining insights into these genomic loci could prove invaluable for enhancing the cultivation of more robust and productive peanut plants in the future.

**Abbreviations**
FBL: first branch length
GO: gene ontology
GWAS: genome‐wide association study
MSH: main stem height
NSK: number of nodes on the main stem
PARMS: penta‐primer amplification refractory mutation system
qRT‐PCR: quantitative real‐time polymerase chain reaction
QTL: quantitative trait locus
SD: stem diameter
SNP: single‐nucleotide polymorphism
WGRS: whole‐genome resequencing

## 1 INTRODUCTION

Peanut (*Arachis hypogaea* L.) is a vital oilseed crop extensively cultivated in over 100 countries, with China being the leading producer, exporter, and consumer of peanut oil (Liao, 2020; Yu, 2011). Despite the significant market demand for peanuts, there is substantial potential to meet this demand through genetic improvements of key agronomic traits (Q. Lu et al., 2024; Varshney et al., 2013). Traits related to peanut architecture are closely associated with yield (Khedikar et al., 2018; Varshney et al., 2013) and may also influence water utilization and diseases resistance in peanuts (Sarkar et al., 2020). In this study, we focus on four traits related to plant architecture: main stem height (MSH), number of nodes on the main stem (NSK), stem diameter (SD) and first branch length (FBL). These traits are quantitative, governed by multiple genetic loci, and influenced by various environmental factors (Cheng et al., 2015; Q. Lu et al., 2024; S.‐Z. Zhang et al., 2023). This complexity presents considerable challenges in developing diagnostic markers for peanut architecture traits. Efforts to address this challenge have been undertaken through quantitative trait locus (QTL) mapping and genome‐wide association study (GWAS) (Chen et al., 2018; L. Huang et al., 2015; Khedikar et al., 2018; Y. J. Li, 2016; Y. Li et al., 2017; Lv et al., 2018; Sarkar et al., 2020; J. Wang et al., 2021; S. Z. Zhang et al., 2019). Although MSH, FBL, and NSK are significantly correlated (Q. Lu et al., 2024; J. Wang et al., 2021), few shared genomic regions have been identified among these traits. Furthermore, the majority of these studies have been conducted with a resolution limited to large‐scale genomic intervals or candidate genes; thus, quantitative trait nucleotides or diagnostic markers with finer resolution remain to be discovered.

High‐throughput sequencing has significantly advanced the QTL analysis of essential agronomic traits in peanut, including yield, fatty acid content, protein content, abiotic stress tolerance, and biotic stress resistance (Y. Li et al., 2017; Guo et al., 2021; Q. Lu et al., 2024; Raza et al., 2024; J. Wang et al., 2024, 2025). GWASs conducted on natural populations, particularly when integrated with high‐throughput sequencing techniques such as whole‐genome resequencing (WGRS), can facilitate the identification of a larger number of significant single‐nucleotide polymorphisms (SNPs). However, the application of WGRS in GWAS analyses concerning traits related to plant architecture in peanuts remains limited.

In the present study, we utilize WGRS data from 160 peanut landraces, which encompass 82.4% of the genetic diversity found in Chinese landraces (Shan & Yan, 2018; P. Yan et al., 2020; H. Zhang, 2013), to conduct GWAS analyses on four traits associated with peanut architecture: MSH, SD, NSK, and FBL. By integrating evidences from our GWAS analysis, previous QTL and GWAS, as well as our (PAMRS) and quantitative real‐time polymerase chain reaction (qRT‐PCR) validation, we aim to achieve a more comprehensive understanding of the genetic basis of peanut architecture.

## 2 MATERIALS AND METHODS

### 2.1 Plant materials and phenotype collection

In this study, we examined 160 Chinese peanut landraces (C. X. Yan et al., 2019). These landraces were cultivated in Laixi, China, from 2020 to 2023 (Table S1). Each accession was grown in two‐row plots consisting of 40–46 plants, measuring 0.80 m in width and 5.00 m in length. Prior to harvest, four peanut height‐related traits were measured for each accession: FBL, NSK, SD, and MSH. Each trait was measured in three to five plants per accession per year, with measurement units for MSH and FBL being centimeters and for SD being millimeter (Jiang et al., 2006). A boxplot was used to show the distributions of the four traits across four consecutive years (Figure S1; Table S2). To minimize environmental influences, a mixed linear model (MLM) was constructed for each trait using the “lme4” package in R (https://www.r‐project.org/). We extracted the best linear unbiased prediction (BLUP) estimates of the random genetic effect for each trait and utilized these BLUP values in subsequent GWAS analyses. We calculated the correlation coefficient for each trait pair using the “cor” function in R (https://cran.r‐project.org/bin/windows/base/) and estimated the broad‐sense heritability (*H* ^2^) for each trait employing the “lme4” package in R.

**Core Ideas**

- We identified 411 significant single‐nucleotide polymorphisms (SNPs) associated with four traits related to peanut architecture through a genome‐wide association study.
- For the trait of stem diameter, we identified a novel genomic region on Arahy.03:39916768–42652757, associated with this trait for the first time.
- Two SNPs located at Arahy.09:112028951 and Arahy.09:112272948 were identified as the potential diagnostic markers for main stem height (MSH) and first branch length (FBL).
- An SNP located at Arahy.05:53493734 was associated with MSH, FBL, and the number of nodes on the main stem. The genomic region surrounding this SNP has been annotated as part of the protein kinase superfamily, which plays a crucial role in plant development.

### 2.2 Whole‐genome SNP identification

WGRS data of 160 peanut landraces are publicly available in the Sequence Read Archive database (the accession number: PRJNA857148). The data were aligned to the reference genome of cultivated peanut (*A. hypogaea* ‘Tifrunner v1’; https://www.peanutbase.org) using BWA v0.7.15, allowing for less than 4% mismatch and a maximum of one gap (Li & Durbin, 2009). SNPs were identified using GATK's Unified Genotyper v4.0 (https://software.broadinstitute.org/gatk). The SNP filtering procedures were as follows: (i) phred score > 2.0, (ii) coverage depth > 5, (iii) missing ratio < 60%, and (iv) a global minor allele frequency > 0.05. The genic and intergenic distributions of all SNPs were determined using ANNOVAR (K. Wang et al., 2010). The linkage disequilibrium (LD) decay analysis and genetic structure analysis of the same population were completed by J. Wang et al. (2025). For details, please refer to the following publication: https://link.springer.com/article/10.1007/s00122‐025‐04923‐x.

### 2.3 Genome‐wide association study

GWAS analysis was performed using TASSEL v5.2.3 to identify SNPs associated with the traits MSH, NSK, SD, or FBL (Bradbury et al., 2007). An MLM was employed to evaluate each SNP sequentially as a fixed effect while concurrently incorporating population structure (fixed effect) and kinship (random effect). The matrix of pairwise kinship coefficients was calculated using SPAGeDi v1.5 (Hardy & Vekemans, 2002). The *Q* matrix, representing the population structure, was estimated by Admixture (Alexander et al., 2009). A triangular correlation heatmap was produced using LDBlockShow v2.6.3 (Dong et al., 2021). Significance in the GWAS was determined using a *p* value threshold of 10^−4^.

### 2.4 Survey of QTLs for peanut architecture‐related traits

To identify candidate genes associated with height‐related traits in peanuts, we conducted a comprehensive literature review of QTL and GWAS related to peanut architecture (Table S3). All genes located within the candidate genomic regions identified by these QTL and GWAS over the past decade were considered as candidate genes. Gene ontology (GO) enrichment analysis and Kyoto Encyclopedia of Genes and Genomes pathway analysis were performed using the OmicShare web server (www.omicshare.com/tools) (D. W. Huang et al., 2009).

### 2.5 Penta‐primer amplification refractory mutation system (PARMS) genotyping

To confirm the GWAS results and detect a significant difference between genotypes, the genotyping of five selected significant SNPs was conducted on 160 samples using PARMS (Gentides) (Table S4) (J. Lu et al., 2020). Primers for this analysis were designed using Primer Premier 5.0. Following DNA extraction from each sample, PCR reactions were set up in 160‐well PCR plates for PARMS genotyping. Each PCR reaction well, with a total volume of 5 µL, included 2× PARMS PCR reaction mix, allele‐specific primers at a concentration of 150 nM each, a locus‐specific primer at 400 nM, and 1.4 µL of the DNA template. To prevent evaporation, 5 µL of mineral oil was added to each well. The thermal cycling conditions for the PARMS protocol began with an initial denaturation step at 95°C for 15 min, followed by 10 cycles of denaturation at 95°C for 20 s and annealing for 1 min, starting at 65°C and decreasing by 0.8°C per cycle till it reached 57°C. The program then continued with 33 cycles of denaturation at 95°C for 22 s, followed by annealing at 57°C for 1 min. The PCR plates were read using a TECAN Infinite M1000 plate reader. SNP detection was performed using the online software SNPdecoder (http://www.snpway.com/snpdecoder/). In each genotyping, three main sample architectures were identified: homozygous samples (majority), heterozygous samples, and samples with negative genotypes. The significant differences between SNP genotypes were assessed using a Student's *t*‐test.

### 2.6 qRT‐PCR verification

To investigate the expression patterns of the selected SNPs, Four higher plant height accessions (cc5: 16 cm, cc100: 18 cm, cc142: 21 cm, and Kangqing 10: 23 cm), four lower plant height accessions (cc51: 78 cm, cc58: 85 cm, cc113: 89 cm, and Yunnan Qicai: 91 cm), three shorter SD accessions (cc94: 4.77 mm, cc100: 4.83 mm, and cc156: 5.27 mm), and three large SD accessions (cc127: 8.92 mm, cc11: 9.50 mm, and cc139: 9.72 mm) were collected in Qingdao in 2024 and 2025. Fresh stems were harvested from three developmental stages: seedling stage (S1), flowering stage (S2), and pod stage (S3). Total RNA was extracted using the EASY spin plant RNA kit (Ailab). Subsequently, all samples were treated with DNase I (Takara), and the RNA concentration was determined using a NanoDrop ND‐1000 (Thermo). The extracted RNA was then reverse transcribed into cDNA using M‐MLV reverse transcriptase, and qRT‐PCR of five candidate genes was performed with the BYBR Premix Ex Taq Kit (Takara) on a Step One System (Applied Biosystems) (Table S5). The qRT‐PCR reaction consisted of an initial denaturation step at 95°C for 10 min, followed by 40 cycles of 95°C for 15 s and 60°C for 30 s. The relative expression levels of each gene were calculated using the 2^−∆∆^ *^Ct^* method, normalizing the gene expression to a reference gene (*Actin*) based on three biological replicates.

## 3 RESULTS

### 3.1 Sequencing and distribution of genetic variations in the peanut genome

A total of 894,250 high‐quality SNPs were identified (Table S6). The majority of these genome‐wide SNPs were found in intergenic regions, accounting for 79.25% of the total. In contrast, SNPs located in exonic and intronic regions, as well as those found upstream and downstream of annotated genes, constituted a smaller proportion of the total at 1.63%, 3.18%, and 15.03%, respectively (Table S7). “A/G” was the most abundant SNP, accounting for 34.10% of the total SNPs, followed by “C/T” (33.55%). Other SNPs, including “A/C,” “G/T,” “A/T,” and “C/G,” contributed 8.69%, 8.96%, 9.46%, and 5.19% of the total SNPs, respectively. The genome‐wide transition/transversion (*T*s/*T*v) ratio for the analyzed peanut genome data was calculated to be 1.79.

### 3.2 Trait correlation and heritability

**FIGURE 1:**
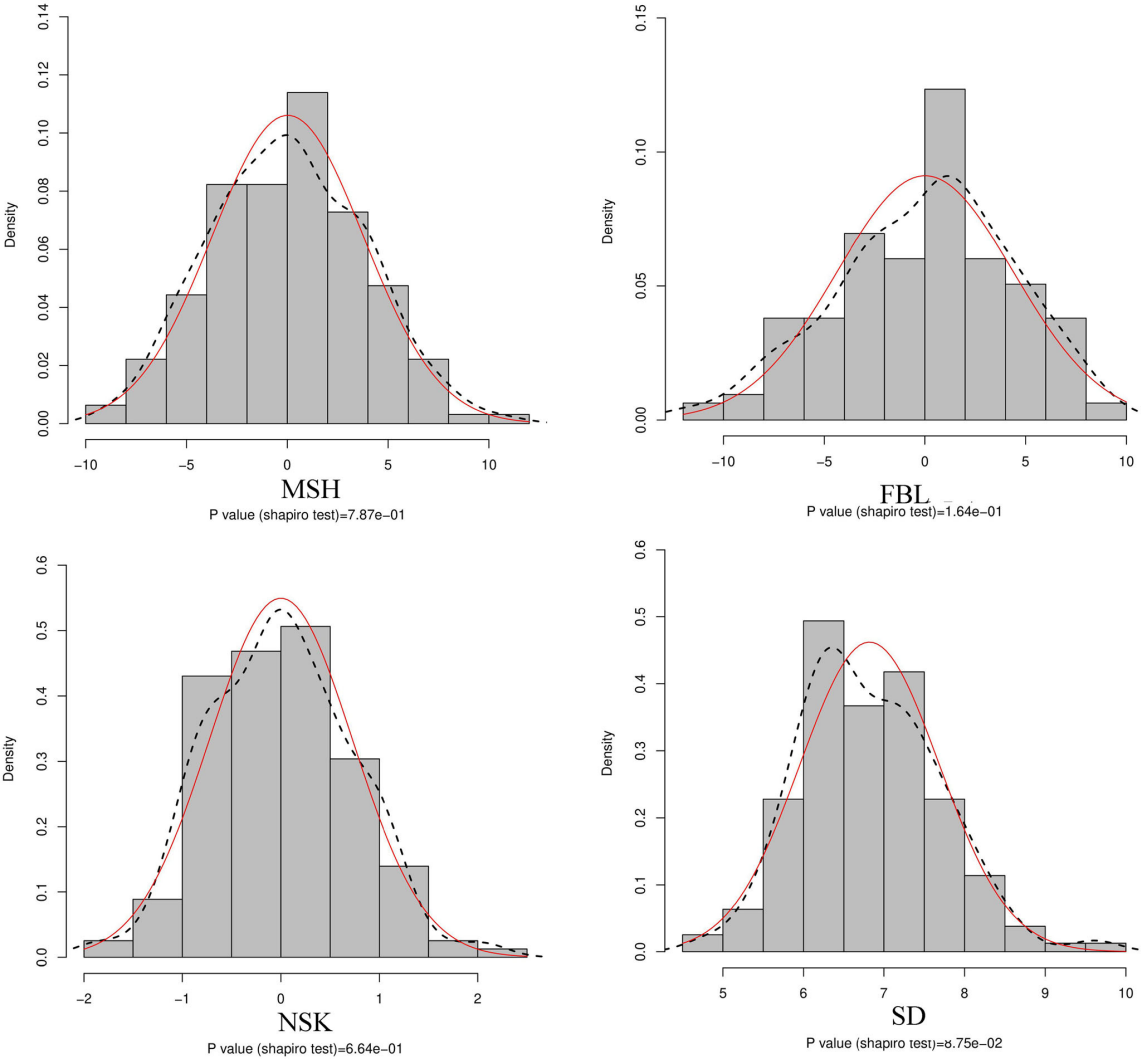
Frequency distribution of the studied peanut architecture‐related traits. *X*‐axis: the best linear unbiased prediction (BLUP) estimates values for each trait. Black dotted line: peanut height density plot; red line: normal distribution. FBL, first branch length; MSH, main stem height; NSK, number of nodes on the main stem; SD, stem diameter.

A total of 160 peanut accessions were phenotyped for MSH, FBL, SD, and NSK under three environmental conditions. All traits exhibited a normal distribution with low skewness and kurtosis values (Figure 1). We observed a medium level of heritability (*H* ^2^: 0.45–0.67) for all traits. Notably, a significant correlation was observed between FBL and MSH (*r *= 0.64), while the lowest correlation was found between NSK and SD (*r *= 0.22) (Table 1; Figure S2).

**TABLE 1:** Summary statistics of the four studied peanut traits.

Traits	Environments	Max.	Min.	Average	SD	CV	H2
SD		9.74	4.79	6.83	0.86	12.71	0.45
	2020	9.99	5.04	7.08	0.87	12.25	
	2021	9.74	4.79	6.83	0.86	12.73	
	2022	9.48	4.53	6.56	0.85	13.13	
	2023	9.74	4.79	6.83	0.86	12.72	
MSH		71.87	22.11	49.18	11.42	23.31	0.67
	2020	76.20	22.27	49.69	12.26	24.60	
	2021	82.27	18.93	50.26	12.57	25.01	
	2022	71.50	22.13	46.21	9.04	19.99	
	2023	68.10	25.11	50.54	11.81	23.63	
FBL		66.15	14.89	36.32	9.64	26.93	0.65
	2020	70.80	10.80	34.62	11.20	32.34	
	2021	64.93	13.87	36.28	9.93	27.50	
	2022	57.30	14.67	34.95	7.96	23.18	
	2023	71.55	20.22	39.41	9.47	24.70	
NSK		26.97	9.36	17.31	3.32	19.38	0.53
	2020	28.62	9.93	17.48	3.49	20.37	
	2021	26.00	7.47	16.56	3.56	21.38	
	2022	28.13	9.47	18.48	3.52	19.48	
	2023	25.11	10.56	16.70	2.70	16.28	

*Note*: The units of measurement used for FBL and MSH are centimeters, and for SD is millimeter.

Abbreviations: CV, coefficient of variance; FBL, first branch length; Max, maximum; Min., minimum; MSH, main stem height; NSK, number of nodes on the main stem; SD, stem diameter.

### 3.3 GWAS in peanut

**FIGURE 2:**
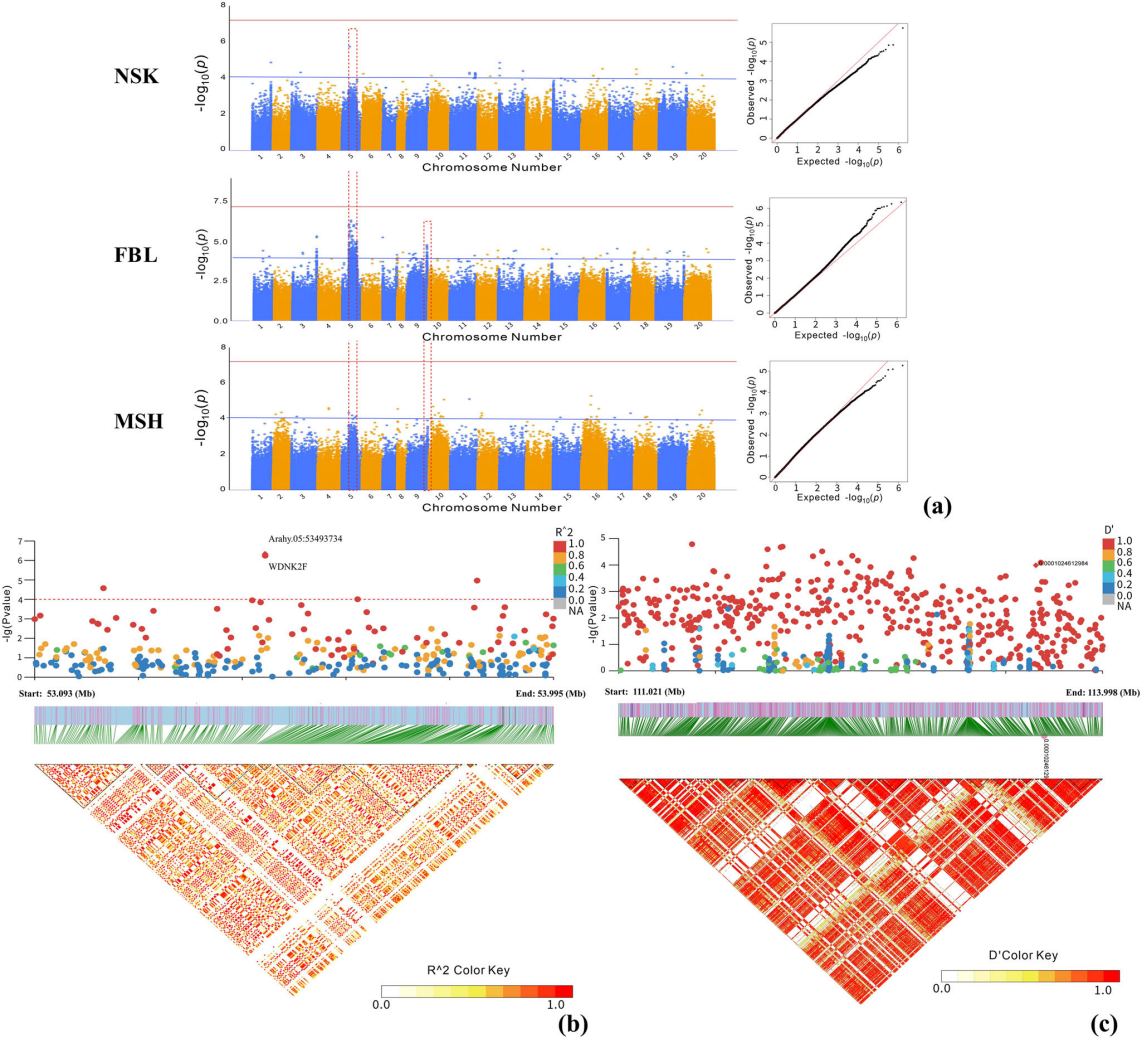
Genome‐wide association study (GWAS) results for first branch length (FBL), main stem height (MSH), and number of nodes on the main stem (NSK). (a) the Manhattan plots and quantile–quantile (*QQ*) plot. The blue and red horizontal lines represent the significance thresholds of −log_10_(*p*) = 4 and −log_10_(*p*) = 7, respectively. (b) Local plots showing the zoomed‐in view of single‐nucleotide polymorphism (SNP) associations on Arahy.05 for MSH. The bottom shows the linkage disequilibrium (LD) heatmap over the peak SNP clusters at 53.093–53.995 Mb on Arahy.05. (c) Local plots showing the zoomed‐in view of SNP associations on Arahy.09 for FBL. The bottom shows the LD heatmap over the peak SNP clusters at 111.021–113.998 Mb. The red dots indicated significant SNPs. FBL, first branch length; MSH, main stem height; NSK, number of nodes on the main stem.

A total of 411 SNPs (*p* < 10^−4^) were significantly associated with the four architecture‐related traits (see Table S8). All significant SNPs were located in intergenic regions. For FBL, the associated peak SNPs were identified on Arahy.01, Arahy.03, Arahy.05, Arahy.07, Arahy.08, and Arahy.11–Arahy.20, with 152 out of the 212 significant SNPs located on Arahy.05. For MSH, a total of 37 associated peak SNPs were discovered on Arahy.02–Arahy.05, Arahy.08, Arahy.10–Arahy.12, Arahy.15–Arahy.18, and Arahy.20. For NSK, the 28 associated peak SNPs were detected on Arahy.01, Arahy.03, Arahy.05, Arahy.06, Arahy.10–Arahy.13, Arahy.16, and Arahy.18–Arahy.20 (Figures 2 and 4). Between the traits FBL and MSH, a total of 16 shared regions were found (Table S9). The genomic region on Arahy.09:111.021–113.998 Mb is the most frequently reported (Table S10) and contains the homologous gene, *Arahy.S06R8C*, which encodes E3 ubiquitin–protein ligase that recognizes specific substrate proteins and attaches ubiquitin molecules to them, marking them for degradation via the ubiquitin–proteasome pathway. Notably, E3 ubiquitin–protein ligase is responsible for the degradation of DELLA protein, a negative regulator of gibberellin signaling that plays a crucial role in promoting plant stem elongation (Blanco‐Tourinan et al., 2020). Another gene from the same region, *Arahy.WY6NTH*, encodes cinnamyl alcohol dehydrogenase, a key enzyme in the synthesis of lignin monomers that catalyzes the conversion of coniferaldehyde to coniferol. Genetic mutations in this gene lead to reduced stem strength, resulting in a dwarfing phenotype (Gong & Xu, 2010). Notably, more than half of the significant SNPs (152/277 > 54.8%) associated with the three traits (MSH, NSK, and FBL) were localized on Arahy.05, where the top five SNPs, boasting the highest ‐log_10_(*p*) values, were annotated for each trait (Table S11). Notably, MSH, NSK, and FBL shared a common SNP (Arahy.05:53493734), near which a gene encoding a member of the protein kinase superfamily is located. This superfamily is crucial in catalyzing protein phosphorylation and serves as a universally conserved regulator of metabolism and cell division (Lehti‐Shiu et al., 2012).

**FIGURE 3:**
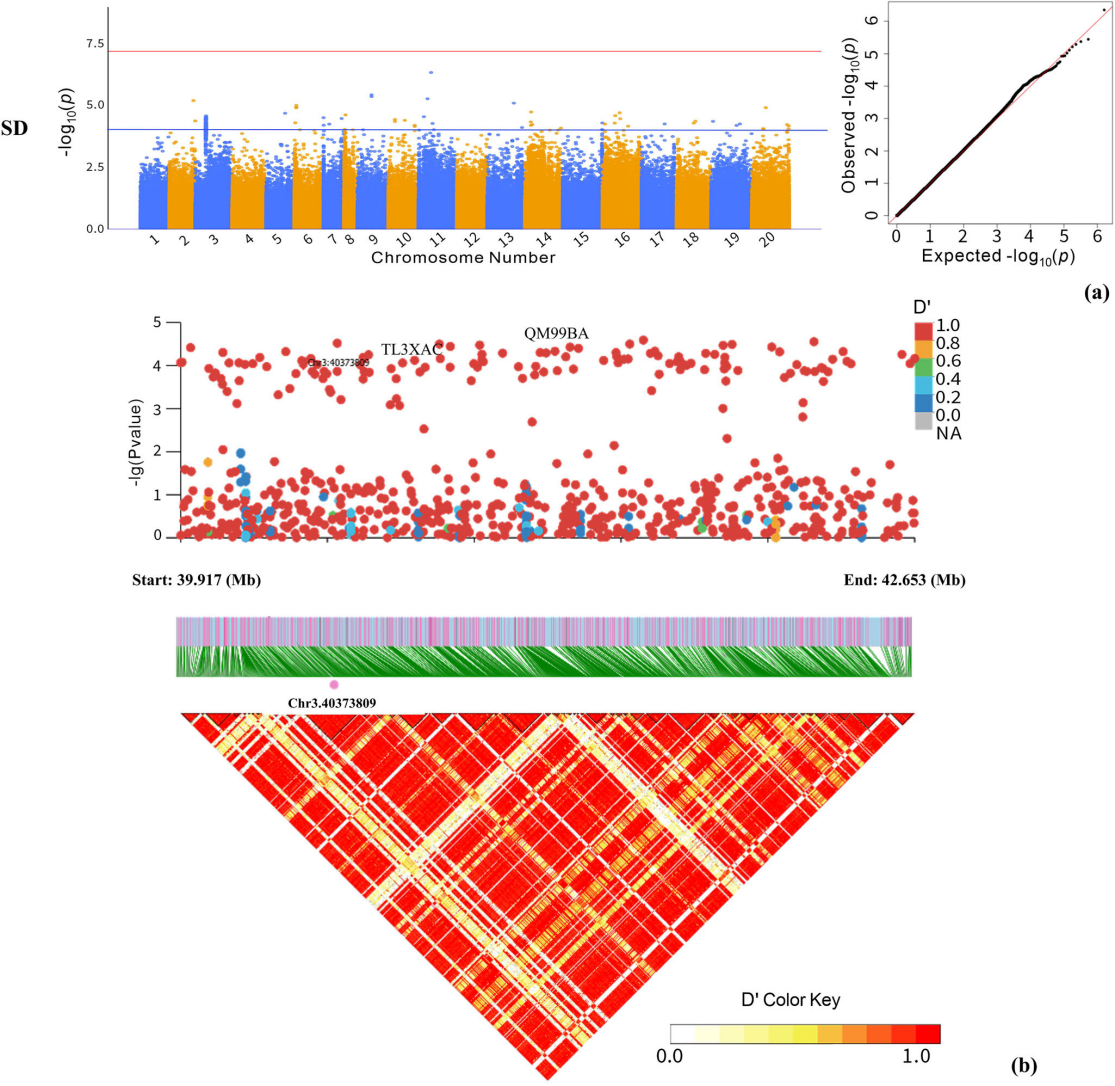
Genome‐wide association study (GWAS) results for stem diameter (SD). (a) the Manhattan plots and quantile–quantile (*QQ*) plot. The blue and red horizontal lines represent the significance thresholds of −log_10_(*p*) = 4 and −log_10_(*p*) = 7, respectively. (b) Local plots showing the zoomed‐in view of single‐nucleotide polymorphism (SNP) associations at Arahy.03:39.917–42.653 Mb for SD. The bottom shows the LD heatmap over the peak SNP clusters. The red dots indicated significant SNPs.

**FIGURE 4:**
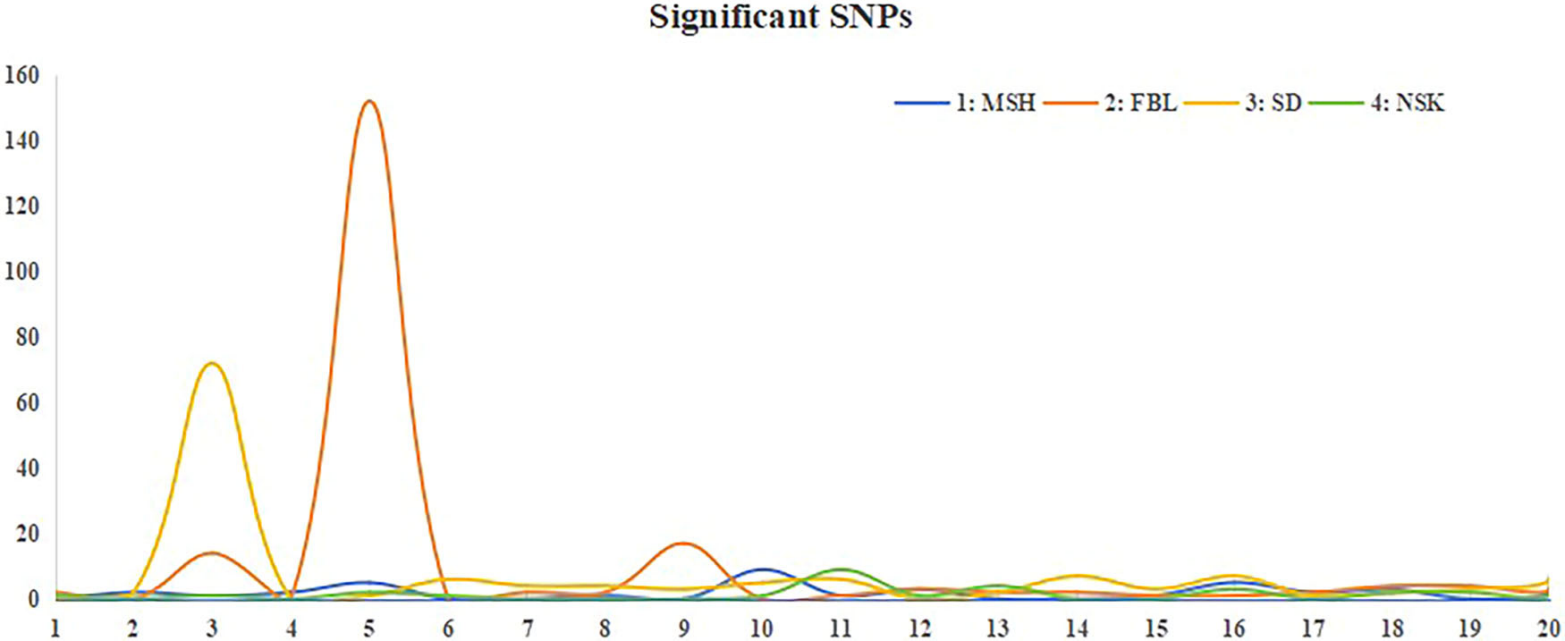
Plot of significant single‐nucleotide polymorphisms (SNPs) for the four studied plant architecture‐related traits. *Y*‐axis: the number of significant SNPs; *X*‐axis: the 20 chromosomes of peanut. FBL, first branch length; MSH, main stem height; NSK, number of nodes on the main stem; SD, stem diameter.

Additionally, for SD, a total of 134 significant SNPs were found on Arahy.02, Arahy.03, Arahy.05–Arahy.11, and Arahy.13–Arahy.20, with more than half of the significant SNPs (72 out of 134, >53.7%) located on Arahy.03:39916768–42652757 (Figures 3 and 4). In this genomic region, a total of 29 functional genes were annotated (Table S12). Based on the studies of homologous functional genes, two candidate genes warrant particular attention. The first is cytokinin (CK) dehydrogenase (*Arahy.QM99BA*), which contains an SNP located at Arahy03:41278968 that is classified as an upstream gene variant. The other gene, *Arahy.TL3XAC*, a member of the ATP‐binding cassette (ABC) transporter I family, contains an SNP located at Arahy03:40619928 that is classified as a downstream gene variant. Furthermore, GO analysis revealed significant overrepresentation of four molecular functions: α‐l‐fucosidase activity, fucosidase activity, primary amine oxidase activity, and CK dehydrogenase activity.

### 3.4 PARMS verification of significant SNP genotypes

To validate the GWAS findings, genotyping was performed on 160 samples using PARMS (Figure 7, 8, 9). The selected SNPs included the shared SNP (Arahy.05:53493774) among FBL, MSH, and NSK; the shared SNPs (Arahy.09:112028951 and Arahy.09:112272948) between FBL and MSH; and two significant SNPs for SD (Arahy.03:41278968 and Arahy.03:40619928). All genotypes obtained through PARMS were largely consistent with those identified via WGRS, showing a high correlation coefficient (*r* > 0.75) (Table 2). According to the student's *t*‐test, the phenotypic differences between genotypes showed significant (*p* < 0.05) at SNP_Arahy.05:53493774, SNP_Arahy.09:112028951, and SNP_Arahy.09:112272948.

**TABLE 2:** Information table for the selected single‐nucleotide polymorphisms (SNPs) that were validated by the penta‐primer amplification refractory mutation system (PARMS) analysis.

Traits/Chr.	SNP position	Annotation	Genotypes No.	Fluorescence of alleles	*p* values (Student's *t*‐test)	Correlation (*r*) between WGRS and PARMS genotyping
**FBL, MSH, and NSK**						
Arahy.05	53493734	WDNK2F (protein kinase superfamily protein) intergenic region	CC(71)/GG(89) High‐/low‐FBL, MSH, and NSK	FAM(78H)/HEX(78L)	0.039/0.043/0.051	0.81
**FBL and MSH**						
Arahy.09	112028951	S06R8C (E3 ubiquitin–protein ligase) intergenic region	GA(7)/GG(67)/AA(76) ‐/Low‐/high‐FBL and MSH	FAM(62H)/HEX(70L)	0.046/0.053	0.85
Arahy.09	112272948	WY6NTH (cinnamyl alcohol dehydrogenase) intergenic region	CT(11)/CC(69)/TT(72) ‐/High‐/low‐FBL and MSH	FAM(66H)/HEX(69L)	0.035/0.041	0.91
**SD**						
Arahy.03	41278968	QM99BA (cytokinin dehydrogenase) upstream gene variant	TT(16)/AA(129) High‐/low‐SD	FAM(18H)/HEX(121L)	>0.05	0.76
Arahy.03	40619928	TL3XAC (ABC transporter I family member) downstream gene variant	TT(21)/CC(128) High‐/low‐SD	FAM(27H)/HEX(121L)	>0.05	0.81

*Note*: Column 4 shows the whole‐genome resequencing (WGRS) genotyping results, while Column 5 shows the PARMS genotyping results.

Abbreviations: ABC, ATP‐binding cassette; FBL, first branch length; MSH, main stem height; NSK, number of nodes on the main stem; SD, stem diameter.

### 3.5 qRT‐PCR verification

The expression patterns of the selected candidate genes associated with the SNPs validated by PARMS (Table 2) were investigated using qRT‐PCR at three developmental stages: seedling stage (S1), flowering stage (S2), and peg tip stage (S3). The expression of *Arahy.WDNK2F*, which was associated with SNP_Arahy.05:53493734, decreases gradually from S1 to S3. Notably, at S1, the expression of *Arahy.WDNK2F* in the group with high peanut height is on average threefold higher than that in the group with lower peanut height (Figure 10).

The expression levels of *Arahy.S06R8C* and *Arahy.WY6NTH* were associated with SNP_Arahy.09:112028951 and SNP_Arahy.09:112272948, respectively. Notably, Arahy.S06R8C exhibited consistently higher expression in the low‐plant‐height group across all three developmental stages, whereas *Arahy.WY6NTH* showed elevated expression in the high‐plant‐height group during the same stages (Figure 11).

The expression levels of *Arahy.QM99BA* and *Arahy.TL3XAC* were associated with SNP_Arahy.03:41278968 and SNP_Arahy.03:40619928, respectively. Notably, the expression of these two genes was higher in accessions with larger SDs at stages S1 and S2. However, at stage S3, the expression of *Arahy.QM99BA* was comparable with larger and shorter SD, while *Arahy.TL3XAC* continued to show higher expression in the larger SD accessions (Figure 12).

## 4 DISCUSSION

The cultivated peanut (*A. hypogaea* L.) is a significant cash crop extensively grown between 45° N and 40° S (Bertioli et al., 2016, 2019; Zhuang et al., 2019). Originating in South America, peanuts and their related species have spread to over 100 countries. Traits associated with peanut architecture are closely linked to yield (Khedikar et al., 2018; Varshney et al., 2013) and may also influence water utilization and disease resistance in peanut (Sarkar et al., 2020). Plant architecture‐related traits, such as MSH, FBL, stem diameter (SD), and NSK, are quantitative and influenced by multiple genes as well as complex environmental factors. In this study, we utilized a WGRS dataset of 160 peanut landraces with rich genetic variation to unveil the genetic underpinning of MSH, FBL, SD, and NSK through GWAS analysis. In contrast to a similar study by J. Wang et al. (2021), which covered only 1.7% of the peanut genome using genotyping by sequencing data, WGRS has identified SNP markers that provide up to 57.49‐fold greater genome coverage, enabling the detection of a larger number of significant SNPs.

**FIGURE 5:**
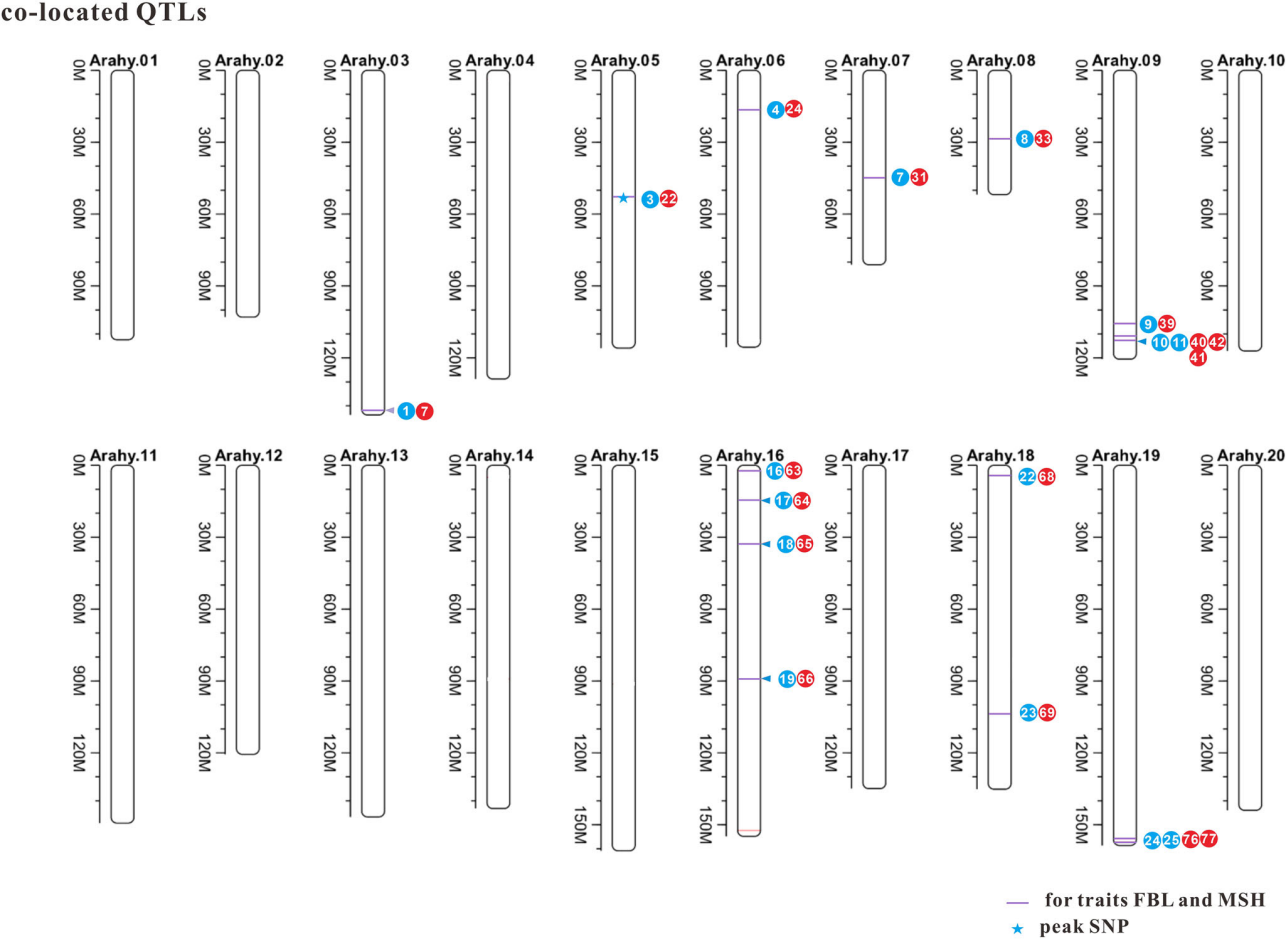
The colocated quantitative trait loci (QTLs) identified to be associated with main stem height (MSH) and first branch length (FBL) from both previous and the present studies. The purple lines represent the co‐localized QTLs; the stars point to a significant single‐nucleotide polymorphism (SNP) (Arahy.05:53493734) identified by the present genome‐wide association study (GWAS) analysis. Red dots signify the QTLs for MSH identified by previous studies, while blue dots indicate the QTLs for FBL that have been identified by previous studies.

**FIGURE 6:**
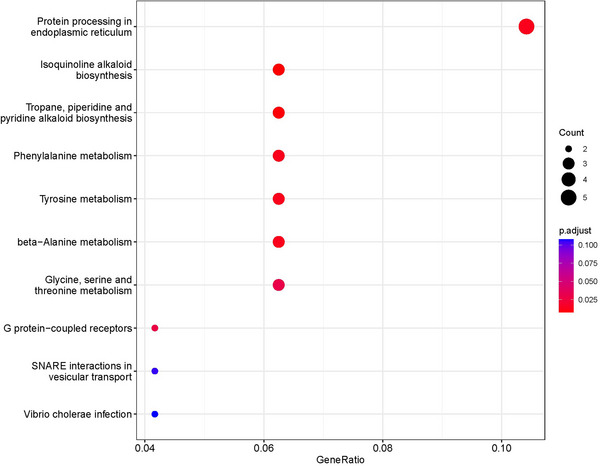
Kyoto Encyclopedia of Genes and Genomes (KEGG) pathway enrichment based on the 176 identified genes.

**FIGURE 7:**
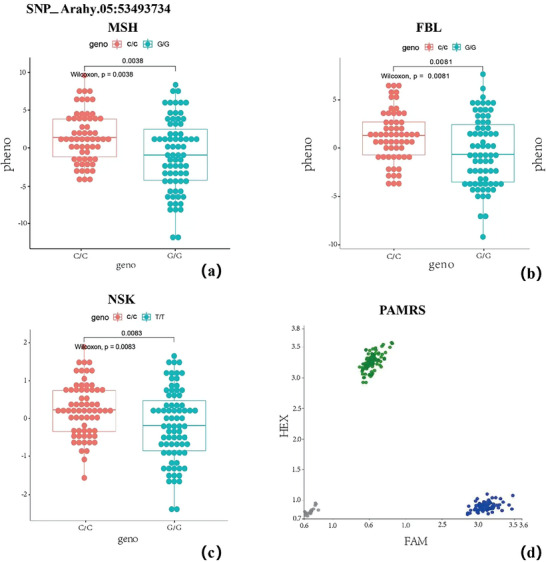
Genotyping results at SNP_Arahy.05:53493734 (where SNP stands for single‐nucleotide polymorphism) for traits main stem height (MSH), first branch length (FBL), and number of nodes on the main stem (NSK). (a–c) Significant difference (*p* value < 0.01) between genotypes identified through whole‐genome resequencing (WGRS). Red dots and green dots represent the two types of nucleotide variation, respectively. (d) Genotyping result validated by PARMS. Green dots: HEX fluorescent signal; blue dots: FAM fluorescent signals; grey dots: negative controls and inconclusive samples. *X*‐axis: the FAM fluorescence signal value/ROS fluorescence signal value; *Y*‐axis: the HEX fluorescence signal value/ROS fluorescence signal value. PAMRS, penta‐primer amplification refractory mutation system.

**FIGURE 8:**
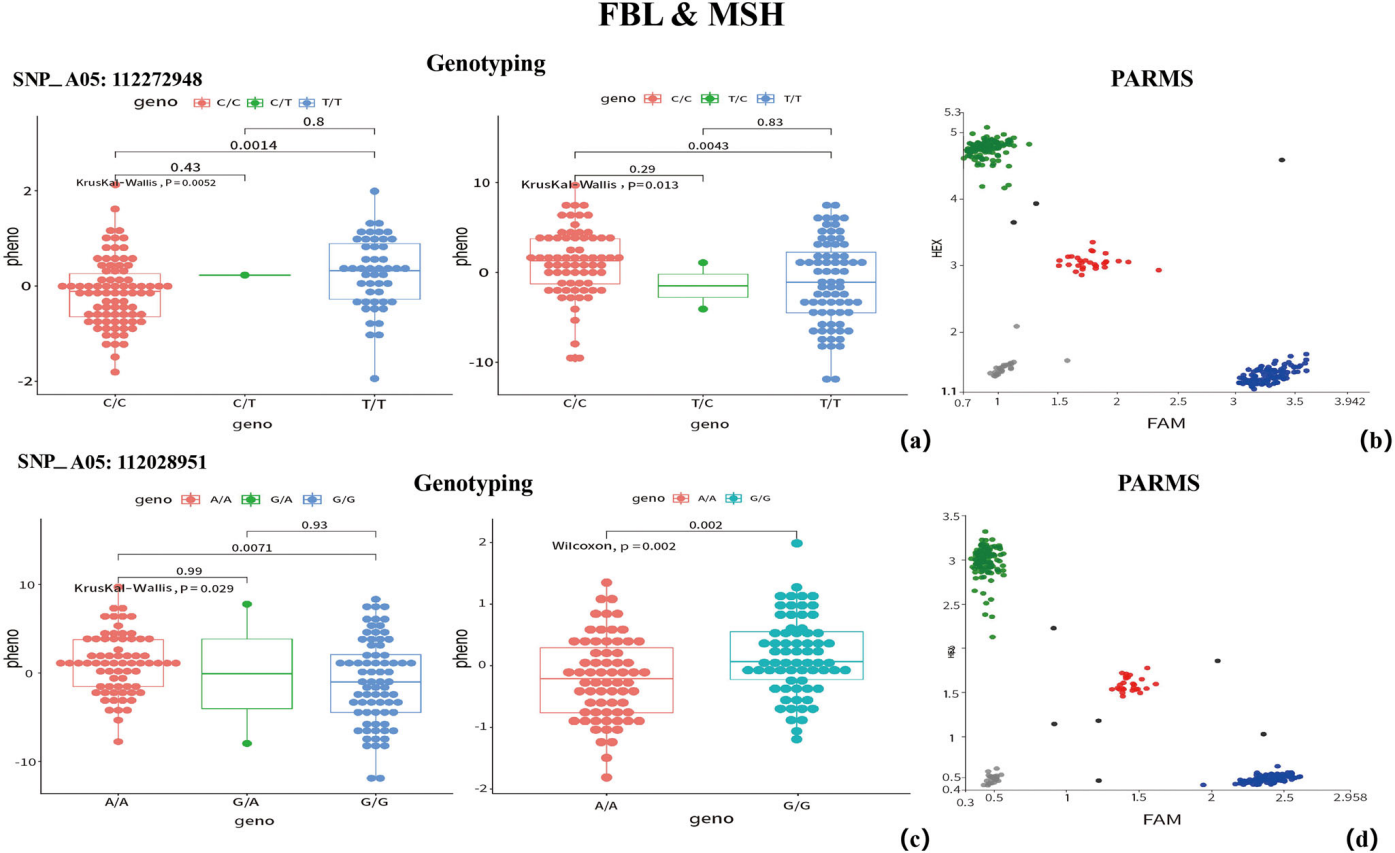
Genotyping at SNP_Arahy.09:112272948 and SNP_Arahy.09:112028951 (where SNP stands for single‐nucleotide polymorphism) for traits first branch length (FBL) and main stem height (MSH). (a, c) Significant differences between the genotypes at the two SNPs (*p* value < 0.05) identified by whole‐genome resequencing (WGRS). (b, d) Genotyping result of these two SNPs obtained using the penta‐primer amplification refractory mutation system (PARMS).

**FIGURE 9:**
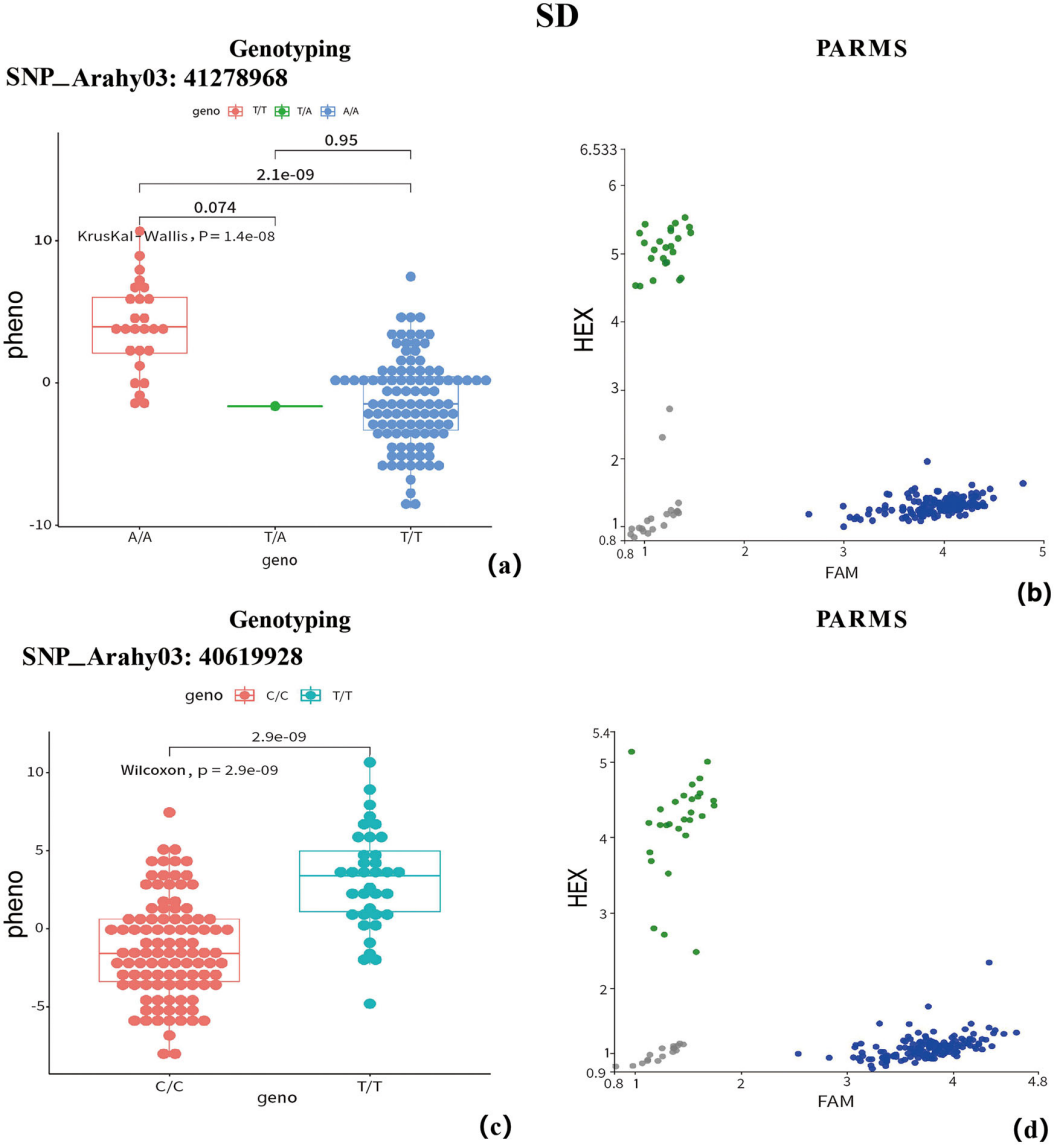
Genotyping at SNP_Arahy.03:41278968 and SNP_Arahy.03:40619928 (where SNP stands for single‐nucleotide polymorphism) for trait stem diameter (SD). (a, c) Significant differences (*p* value < 0.01) between the genotypes at the two SNPs identified by whole‐genome resequencing (WGRS); (b) Genotyping result of these two SNPs obtained using the penta‐primer amplification refractory mutation system (PARMS).

**FIGURE 10:**
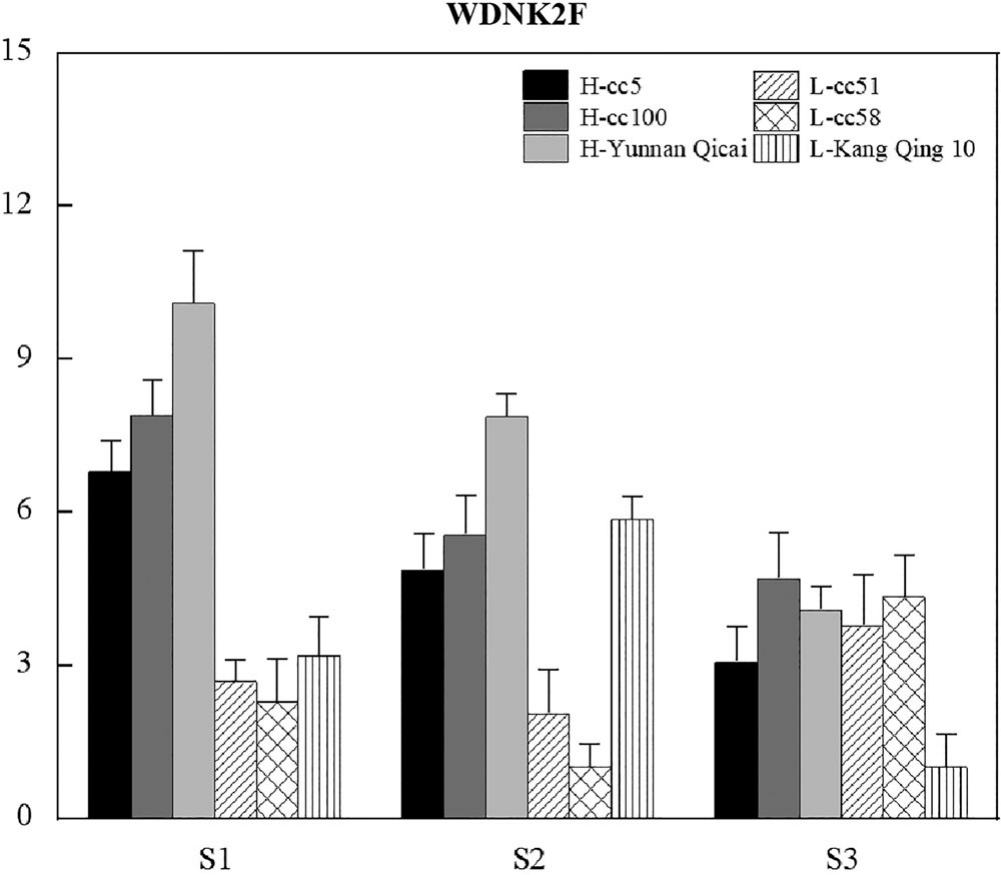
Comparison of the relative gene expression between the high‐ and low‐plant‐height accessions. The gene expression levels were obtained with quantitative real‐time polymerase chain reaction (qRT‐PCR). High‐plant‐height accessions include cc5, cc100, and Yunnan Qicai, while low‐plant‐height accessions include cc51, cc58, and Kangqing 10. S1: seedling stage, S2: flowering stage, and S3: pod stage. The expression levels of *Arahy.S06R8C* and *Arahy.WY6NTH* were associated with SNP_Arahy.09:112028951 and SNP_Arahy.09:112272948 (where SNP stands for single‐nucleotide polymorphism), respectively. Notably, *Arahy.S06R8C* exhibited consistently higher expression in the low‐plant‐height group across all three developmental stages, whereas *Arahy.WY6NTH* showed elevated expression in the high‐plant‐height group during the same stages.

**FIGURE 11:**
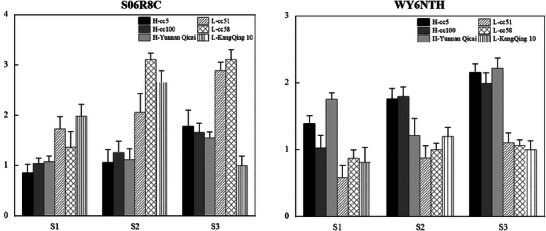
Comparison of the relative gene expression between the high‐ and low‐plant‐height accessions. The gene expression levels were obtained with quantitative real‐time polymerase chain reaction (qRT‐PCR). High‐plant‐height accessions include cc5, cc100, and Yunnan Qicai, while low‐plant‐height accessions include cc51, cc58, and Kangqing 10. S1: seedling stage, S2: flowering stage, and S3: pod stage. The expression levels of *Arahy.QM99BA* and *Arahy.TL3XAC* were associated with SNP_Arahy.03:41278968 and SNP_Arahy.03:40619928 (where SNP stands for single‐nucleotide polymorphism), respectively. Notably, the expression of these two genes was higher in accessions with larger SDs at stages S1 and S2. However, at stage S3, the expression of *Arahy.QM99BA* was comparable with larger and shorter SD, while *Arahy.TL3XAC* continued to show higher expression in the larger SD accessions.

**FIGURE 12:**
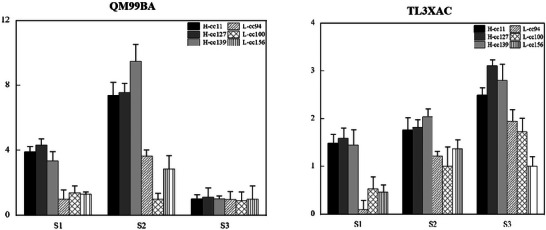
Comparison of the relative gene expression between the larger‐ and shorter‐stem diameter (SD) accessions. The gene expression levels were obtained with quantitative real‐time polymerase chain reaction (qRT‐PCR). Large SD accessions include cc127, cc11, and cc139, while short SD accessions include cc94, cc100, and cc156. S1: seedling stage, S2: flowering stage, and S3: pod stage.

In this study, we identified between 28 and 212 SNPs significantly associated with four peanut traits under study, with over one‐third of these SNPs located on Arahy.05. Many of these SNPs were common to both FBL and MSH, a finding consistent with the strong correlation between the two traits (*r *= 0.64). Additionally, we conducted an extensive literature review, identifying 17 QTLs and GWAS that investigated MSH and FBL using various peanut accessions reported in the last 10 years (Figure 5; Table S3). By integrating these previous findings with our current research, we identified a total of 80 genomic regions associated with MSH and 25 genomic regions associated with FBL (Figures S3 and S4). Among these regions, 16 were found to co‐localize between MSH and FBL (Table S9). Specifically, one co‐localized region was identified on each of chromosomes Arahy.03, Arahy.05, Arahy.06, Arahy.07, and Arahy.08; three on Arahy.09; four on Arahy.16; and two on each of Arahy.18 and Arahy.19 (Figure 5). Within these co‐localized genomic regions, a total of 176 genes were annotated, which were significantly enriched in eight pathways. These pathways include protein processing in the endoplasmic reticulum; isoquinoline alkaloid biosynthesis; tropane, piperidine, and pyridine alkaloid biosynthesis; phenylalanine metabolism; tyrosine metabolism; β‐alanine metabolism; G protein‐coupled receptor signaling; and glycine, serine, and threonine metabolism (Figure 6, 7, 8, 9, 10, 11, 12). Notably, β‐alanine metabolism plays a crucial role in protecting plants from various abiotic and biotic stresses (Klapheck et al., 1988; Moran et al., 2000; Parthasarathy et al., 2019), and our previous research highlighted the enrichment of β‐alanine metabolism in candidate genes for MSH and FBL traits (J. Wang et al., 2021). Additionally, β‐alanine metabolism has been shown to be significantly enriched in differentiating genes between tall and dwarf peanut cultivars (S.‐Z. Zhang et al., 2023). Furthermore, the phenylalanine metabolism pathway was emphasized due to its association with peanut height as reported by Zang et al. (2023) based on transcriptome and metabolome analyses. This pathway also represents a key component of secondary metabolism in plants. GO analysis revealed that the overlapping genes were significantly overrepresented within the tRNA methyltransferase complex, classified under the “Cellular Component” category. The most significant “Biological Processes” identified were the response to azide and the cellular response to azide; azide functions as an inhibitor of electron transport systems (Wilson & Chance, 1967). The most notable “Molecular Function” was diamine oxidase activity, which plays a role in plant stress resistance (Moschou, 2018; Panagiotis, 2017) (Tables S13 and S14).

Although numerous shared candidate genomic regions between FBL and MSH have been identified, there have been no QTL or GWAS to date focusing on NSK or SD in peanuts. In our current study, we identified a significant genome region (Arahy.03:39916768–42652757) containing 72 significant SNPs for SD, which exhibited only a weak correlation with the other three traits. Two annotated genes from this region were identified based on GO term annotation and gene function description. The first gene is CK dehydrogenase (*Arahy.VQM99BA*), coding for a key enzyme that regulates CK homeostasis and plays a crucial role in plant growth and development through the irreversible degradation of CK (Ashikari et al., 2005; Tanabe et al., 2005; Tang et al., 2022; J. Wang et al., 2021). The second gene, *Arahy.TL3XAC*, encodes an ABC transporter. ABC transporter proteins utilize ATP hydrolysis to provide energy for various biological processes. These transporters have been associated with plant seed size and seed fat content, as reported by Kim et al. (2018) and Zhao et al. (2023).

More importantly, we identified an SNP at position Arahy.05:53493734 that is common to three of the studied peanut traits: FBL, MSH, and NSK. A gene located near this peak SNP encodes a member of the protein kinase superfamily, which plays important roles in numerous cellular and biological processes, including cell expansion, seed germination, plant development, pollen tube growth, and responses to abiotic and biotic stressors (Lehti‐Shiu & Shiu, 2012). Besides, this shared SNP resides within a genomic region previously reported by Khedikar et al. (2018) to be associated with plant height, yield per hectare, hundred seed weight, and shelling percentage (Figure 5).

Additionally, two significant SNPs, SNP_Arahy.03:41278968 and SNP_Arahy.03:40619928, associated with SD, are located in the upstream/downstream regions of *Arahy.QM99BA* and *Arahy.TL3XAC*, respectively. Our qRT‐PCR analysis revealed a noticeable difference in gene expression between accessions with high and low SD. The observed differences in expression levels of these genes might be attributed to genetic variation at SNP_Arahy.03:41278968 and SNP_Arahy.03:40619928. However, further verification is needed in future work.

## AUTHOR CONTRIBUTIONS

**Juan Wang**: Data curation; formal analysis; investigation; methodology; software; validation; visualization; writing—original draft; writing—review and editing. **Dachuan Shi**: Data curation; investigation; methodology; validation; visualization; writing—review and editing. **Cuiling Yuan**: Data curation; writing—review and editing. **Yifei Mou**: Data curation; investigation; software; validation; visualization; writing—review and editing. **Haocui Miao**: Resources; validation; visualization. **Yuan Li**: Data curation; writing—review and editing. **Shihua Shan**: Project administration; resources; software; supervision; validation; writing—review and editing.

## CONFLICT OF INTEREST STATEMENT

The authors declare no conflicts of interest.

## Supporting information




**Figure S1**: Boxplot showing the distribution of traits MSH, FBL, NSK, and SD across four consecutive years.

**Figure S2**: Correlations between the studied peanut traits. Dot color and size both represent the degree of correlation. These values represent coefficient of correlation (*r*).

**Figure S3**: QTLs identified to be associated with FBL in both previous and the present studies. Blue cycles represent different QTLs on different chromosomes; Blue star points to the peak SNP regions detected by the present GWAS analysis.

**Figure S4**: QTLs identified to be associated with MSH in both preivous and the present studies. Red cycles represent different QTLs on different chromosomes; Red star points to the peak SNP regions detected by the present GWAS analysis.

**Table S1**: Basic information about the 160 studied peanut accessions.

**Table S2**: The original phenotypic data of MSH, FBL, NSK and SD.

**Table S3**: Information on preivously identified QTLs for MSH and FBL.

**Table S4**: Information table for the primer used in the PARMS analysis.

**Table S5**: Information table for the primers used by qRT‐PCR.

**Table S6**: SNP summary after different filtering steps.

**Table S7**: A summary of the acquired SNPs and InDels in different genic and intergenic regions.

**Table S8**: The distribution of the significant SNPs on each of the 20 chromosomes.

**Table S9**: A summary of the shared candidate genomic regions for MSH and FBL acquired from recent studies.

**Table S10**: A total of 15 candidate genes annotated and GO enrichment on Arahy.09: 111.021‐113.998 Mb for FBL and MSH.

**Table S11**: The top five SNPs on Arahy.05 that were associated with the studied traits.

**Table S12**: A total of 29 candidate genes annotated and GO enrichment on Arahy.03: 39916768–42652757 for SD.

**Table S13**: GO enrichment of the 176 genes in the overlapped genomic regions (*p*.adjust < 0.05).

**Table S14**: KEGG pathway analysis of the 176 genes in the overlapped genomic regions (*p*.adjust < 0.05).

## Data Availability

The original contributions presented in the study are included in the article/Supporting Information; further inquiries can be directed to the corresponding author/s.
