# Supplementary figures and images for: Genome‐wide identification of quantitative trait nucleotides for plant architecture‐related traits in peanut
Source: Plant Genome. 2025 Nov 3;18(4):e70119. doi: 10.1002/tpg2.70119 (PMC12583921; doi:10.1002/tpg2.70119)

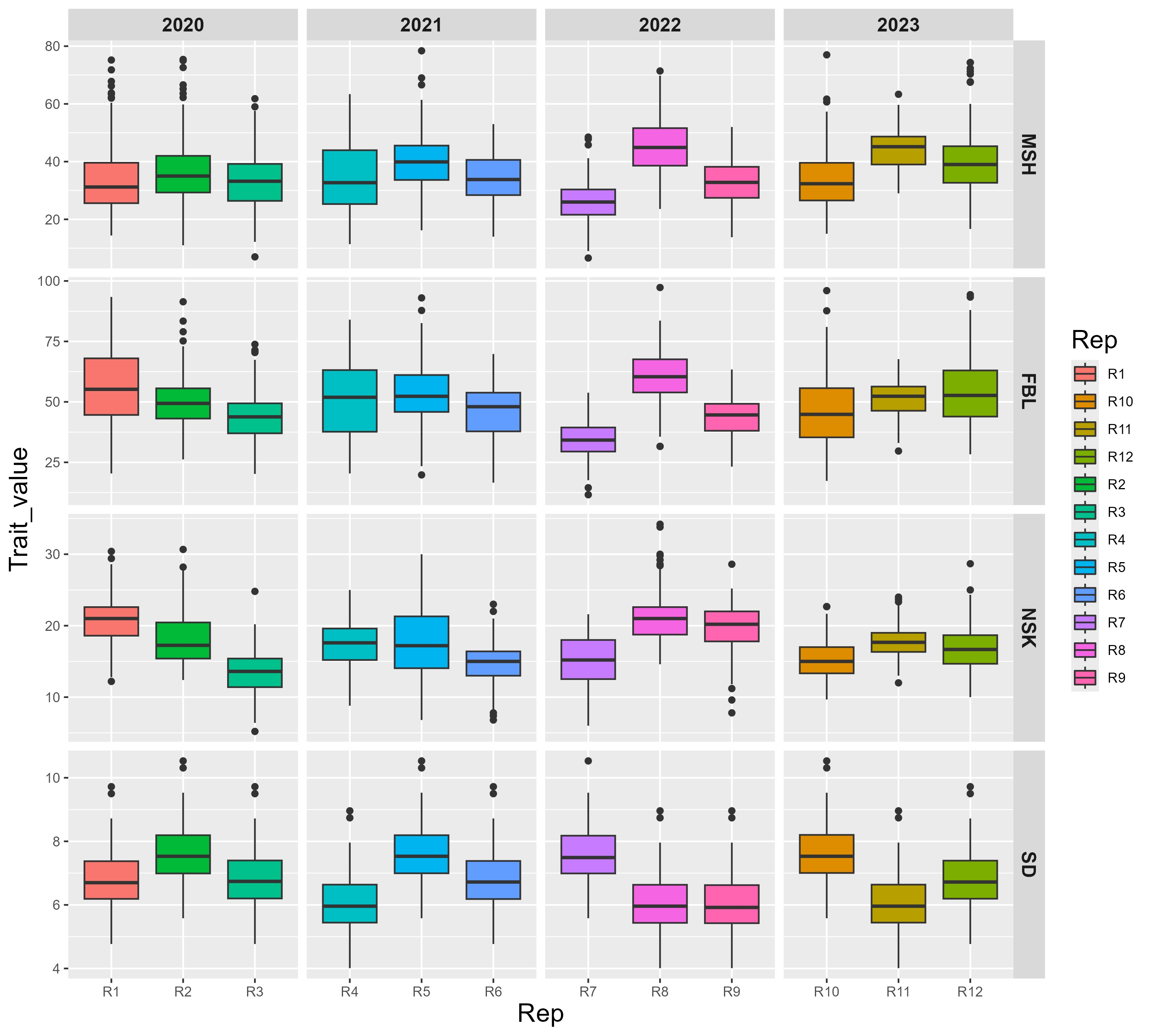

Supplement: Supplementary file 1 — Figure S1: Boxplot showing the distribution of traits MSH, FBL, NSK, and SD across four consecutive years. [file TPG2-18-e70119-s004.jpg]

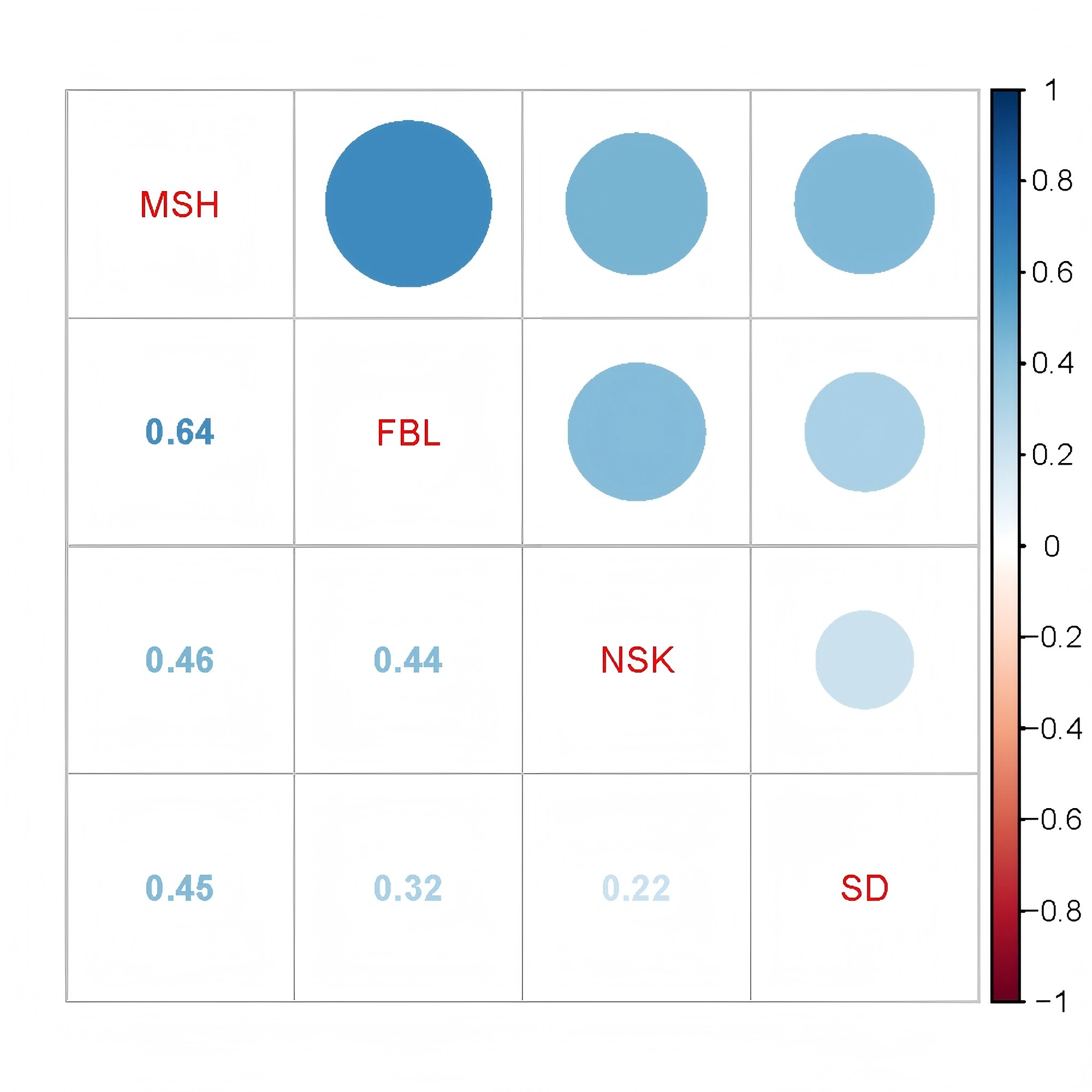

Supplement: Supplementary file 2 — Figure S2: Correlations between the studied peanut traits. Dot color and size both represent the degree of correlation. These values represent coefficient of correlation (r). [file TPG2-18-e70119-s015.jpg]

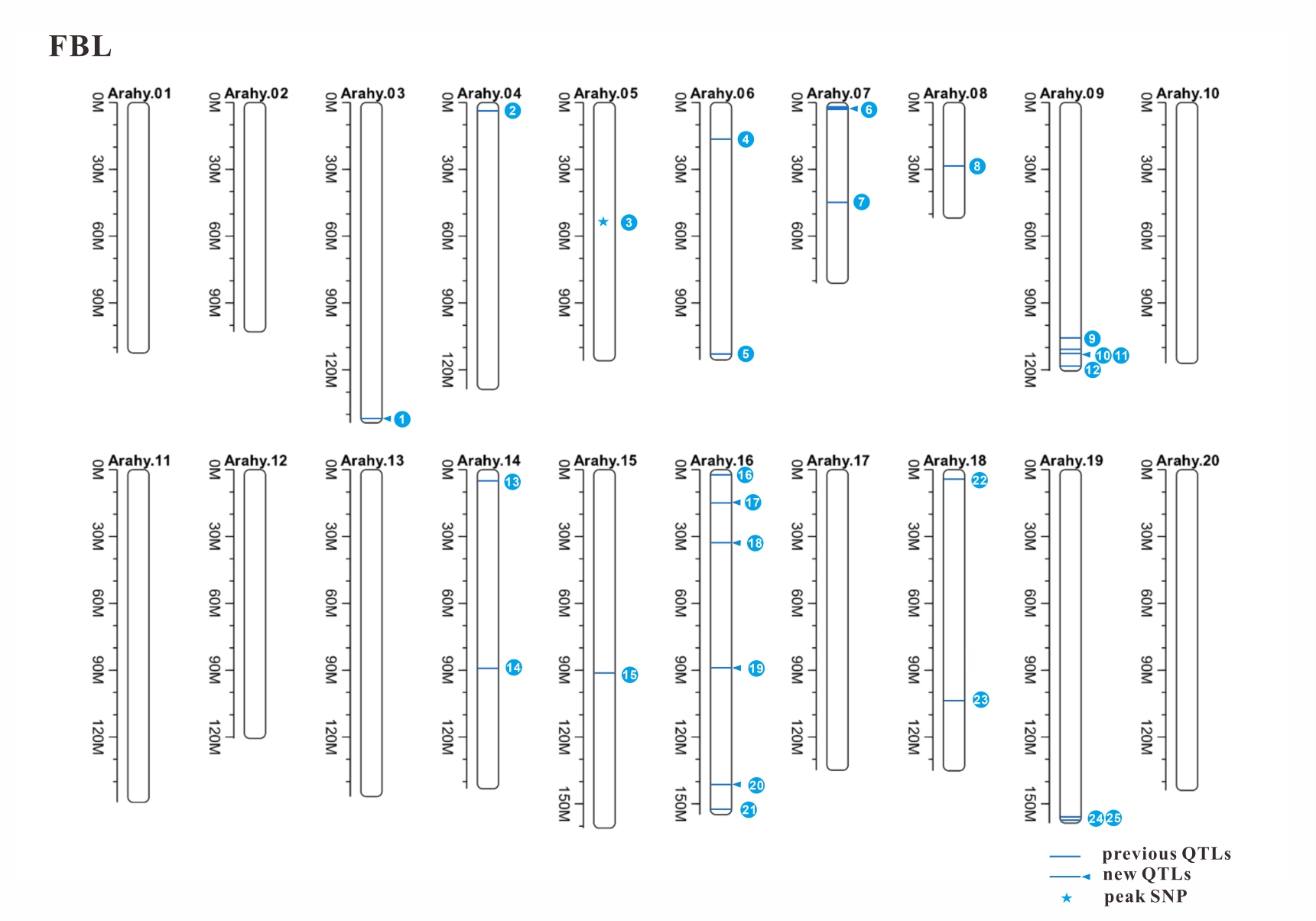

Supplement: Supplementary file 3 — Figure S3: QTLs identified to be associated with FBL in both previous and the present studies. Blue cycles represent different QTLs on different chromosomes; Blue star points to the peak SNP regions detected by the present GWAS analysis. [file TPG2-18-e70119-s009.jpg]

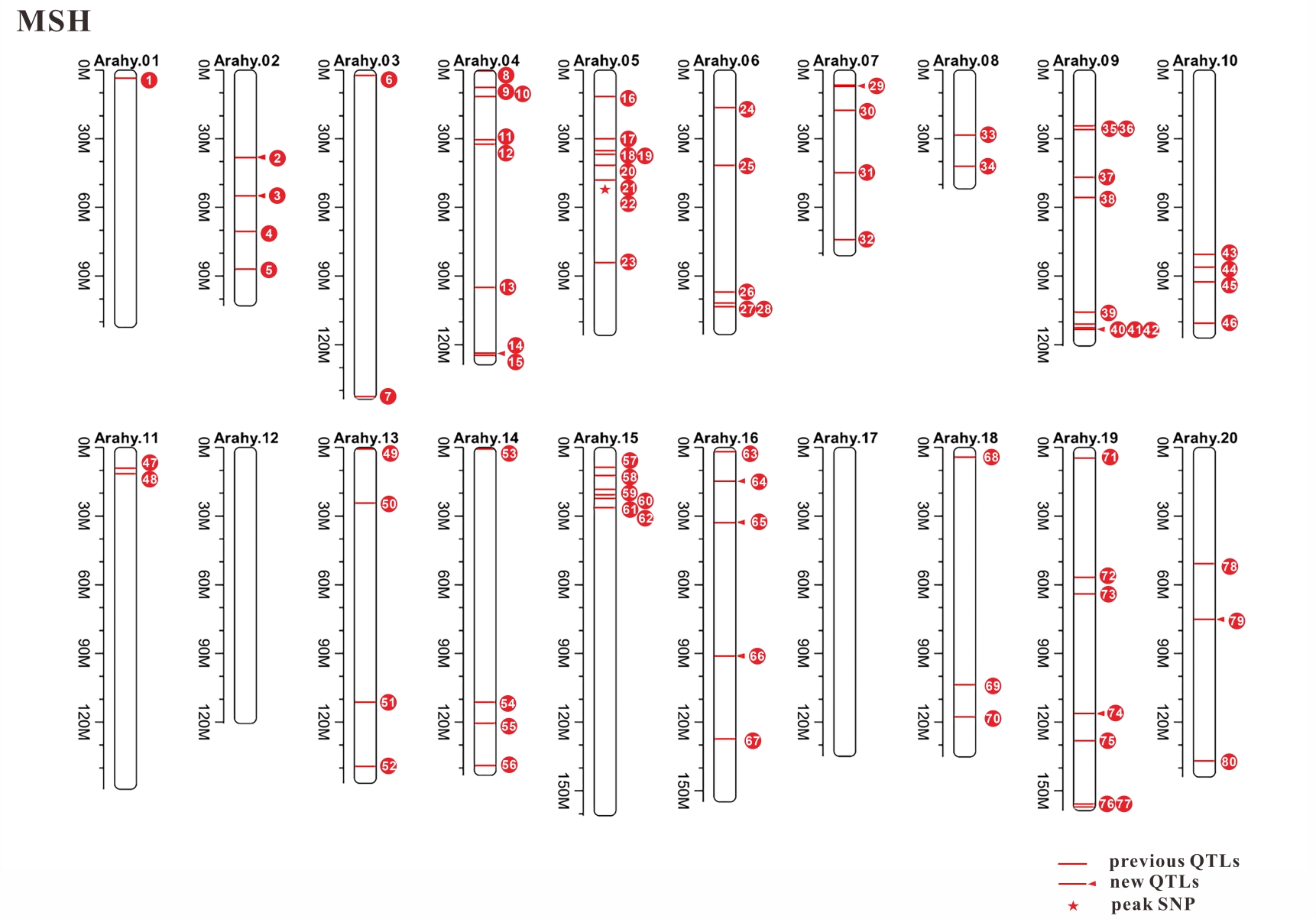

Supplement: Supplementary file 4 — Figure S4: QTLs identified to be associated with MSH in both preivous and the present studies. Red cycles represent different QTLs on different chromosomes; Red star points to the peak SNP regions detected by the present GWAS analysis. [file TPG2-18-e70119-s013.jpg]
